# The effects of motion on the dose distribution of proton radiotherapy for prostate cancer

**DOI:** 10.1120/jacmp.v13i3.3639

**Published:** 2012-05-10

**Authors:** Sima Qamhiyeh, Dirk Geismar, Christoph Pöttgen, Martin Stuschke, Jonathan Farr

**Affiliations:** ^1^ Westdeutsches Protonentherapiezentrum Essen Essen Germany; ^2^ Klinik für Strahlentherapie Universitätsklinikum Essen Essen Germany

**Keywords:** prostate cancer, treatment planning, particle therapy, robustness, retrospective study

## Abstract

Proton radiotherapy of the prostate basal or whole seminal vesicles using scattering delivery systems is an effective treatment of prostate cancer that has been evaluated in prospective trials. Meanwhile, the use of pencil beam scanning (PBS) can further reduce the dose in the beam entrance channels and reduce the dose to the normal tissues. However, PBS dose distributions can be affected by intra‐ and interfractional motion. In this treatment planning study, the effects of intra‐ and interfractional organ motion on PBS dose distributions are investigated using repeated CT scans at close and distant time intervals. The minimum dose ($D_{\text{min}}$) and the dose to 2% and 98% of the volumes ($D_{2\%}$ and $D_{98\%}$), as well as EUD in the clinical target volumes (CTV), is used as measure of robustness. In all patients, $D_{98\%}$ was larger than 96% and $D_{2\%}$ was less than 106% of the prescribed dose. The combined information from $D_{\text{min}}$, $D_{98\%}$ and EUD led to the conclusion that there are no relevant cold spots observed in any of the verification plans. Moreover, it was found that results of single field optimization are more robust than results from multiple field optimizations.

PACS numbers: 87.55.D‐, 87.55.de, 87.53.Bn, 87.55.dk, 87.55.ne

## I. INTRODUCTION

Locally advanced prostate cancers show a strong dose‐response relationship. Proton radiotherapy to the prostate and basal or whole seminal vesicles with a dose up to 82 ${\text{Gy}}_{1.1}$ was found effective and tolerable in retrospective and prospective trials.^(^ ^1^ ^–^ ^3^ ^)^ ${\text{Gy}}_{1.1}$ is the dose in units of Cobalt equivalent doses assuming a radiobiological effect of 1.1.^(^ ^4^ ^)^ The above clinical studies were performed with classic double scattering proton therapy systems. Compared to intensity‐modulated radiation therapy (IMRT), double scattering proton techniques result in better distributions of the low and intermediate dose region to the surrounding organs at risk. The proton dose distributions are similar or better in the high‐dose region, depending on the lateral penumbra of the proton beam.^(^ ^5^ ^–^ ^6^ ^)^ The applied dose distributions have similar dependencies on interfractional anatomic motions as dose distributions by IMRT.^(^ ^7^ ^)^ According to existing planning studies,^(^ ^8^ ^–^ ^10^ ^)^ intensity‐modulated proton radiotherapy (IMPT) using pencil beam scanning techniques (PBS) can further improve the dose distributions achieved by passively scattered protons, and can give superior dose distributions than current IMRT techniques. However, inter‐ and intrafractional motion might affect PBS dose distributions to larger extent.^(^ ^10^ ^–^ ^12^ ^)^

This paper is a retrospective study which is carried out with repeated planning CT scans to estimate the effect of intra‐ and interfractional organ motion on the resulting dose distribution. It focuses on intermediate‐ to high‐risk prostate cancer treatment using PBS. In the study, the target volume coverage and dose to the organs at risk were assessed for four different treatment planning setups. In contrast to a robustness study which focuses only on interfractional motion effects,^(^ ^12^ ^)^ the present study distinguishes between intra‐ and interfractional motion, and analyses the effects on dose distribution in clinical prostate cases.

## II. MATERIALS AND METHODS

All patients who were considered for the study have been treated with either 3D conformal or IMRT between December 2008 and March 2009 at our institution. Before their treatment, the patients received a planning CT (CT‐A1) and two low‐dose CTs (CT‐A2 and CT‐A3). The CTs were taken five minutes (CT‐A2) and ten minutes (CT‐A3) after the reference planning CT (CT‐A1) and during the same immobilization session. A second planning CT (CT‐B1) was taken on a second day. The scan protocol used for the low‐dose CTs is identical to the one used for planning CT, except that the mAs used for low‐dose CT is ~30% of the mAs used for the planning CT. The calibration of CT numbers to electron density was the same for both planning and low dose CTs. The multiple CTs were performed to construct the internal target volume of the prostate and seminal vesicles by comparing the different planning CTs using gold markers implanted into the prostate or intraprostatic calcifications. Image guidance by internal prostate markers can effectively decrease the effect of interfractional motion on the coverage of the target by the dose distribution.^(^ ^11^ ^,^ ^13^ ^–^ ^14^ ^)^ The only selection criterion was that all patients had internal prostate markers used for patient repositioning. This resulted in a heterogeneous group of patients in terms of patient positioning aid. In a subset of the patients, where calcifications in the prostate were observed, the calcifications were used as internal markers. In the remaining patients, radio‐opaque prostate implants were implanted at least one week before the first planning CT. The patients were immobilized in the supine position using either a body form or a knee‐foot fixation. The patients were also instructed to empty their bladder and rectum one hour before the acquisition of the CT, and to drink 350 ml of water. In some patients, rectal balloons were used to reduce rectal toxicity and enhance prostate immobilization.^(^ ^15^ ^–^ ^16^ ^)^ The interfractional displacement of the prostate can be considerably high as a result of using the endorectal balloon.^(^ ^17^ ^)^ A rectal balloon (Rüsch AG, Kernen, Germany) of 7 cm length and a 75 ml maximum volume capacity and 30 cm long catheter was used during this study. The heterogeneous patient group was useful to demonstrate the application of the beam arrangements on a variety of clinical conditions.

The clinical target volumes (CTVs), the planning target volumes (PTVs), and the critical organs at risk (OARs) were delineated based on recommendations of the EORTC Radiation Oncology Group.^(^ ^18^ ^)^ For this planning study, two CTV structures were delineated for each patient: CTV1 and CTV2. CTV1 represented the prostate and bilateral seminal vesicles with a margin of up to 0.5 cm around the prostate but not across anatomic borders (i.e., muscles at the outer rectal wall). CTV2 represented the prostate and proximal seminal vesicles within the first 0.5−1.0cm. These CTVs were compatible with the RTOG0415 protocol on IMRT for prostate cancer, and the results of internal motion studies after online correction using implanted markers by Meijer et al., van der Wielen et al., and Kotte et al.^(^ ^19^ ^–^ ^21^ ^)^ In this study, the PTVs were created by expanding the respective CTVs by 0.8 cm around the seminal vesicles and 0.5 cm around the prostate.

The dose prescriptions were 60 ${\text{Gy}}_{1.1}$ for PTV1. An additional 18 ${\text{Gy}}_{1.1}$ was prescribed for PTV2. The summed dose will add to 78 ${\text{Gy}}_{1.1}$ in the prostate. The treatment planning constraints listed in Table 1 include minimum dose ($D_{\text{min}}$), average dose ($D_{\text{mean}}$), maximum dose ($D_{\max}$), dose to a relative volume such as $D_{98\%}$, and volume at given doses (${\text{Gy}}_{1.1}$) such as $V_{82}$ which could be extracted from the dose volume histograms (DVH). All patients had to fulfill all the constraints in Table 1 in all plans. The dose was to be delivered in 2 ${\text{Gy}}_{1.1}$ fractions. It was estimated that the time required to deliver the dose is ten minutes per fraction. Only one fraction would be delivered per day. The dose planned for PTV1 (60 ${\text{Gy}}_{1.1}$) would be delivered first, followed by PTV2 (18 ${\text{Gy}}_{1.1}$).

**Table 1:** Treatment planning constraints: dose volume constraints which should be fulfilled by all initial treatment

	*Dose Constraints* $\left(Gy_{1.1}\right)$					*%Volume Constraints at Given Dose* $\left(Gy_{1.1}\right)$							
*Volume*	$D_{\min}$	$D_{\max}$	$D_{\mathrm{mean}}$	$D_{98\%-}D_{2\%}$	$V_{82}$	$V_{80}$	$V_{75}$	$V_{70}$	$V_{65}$	$V_{60}$	$V_{50}$	$V_{40}$	$V_{10}$
CTV1	60.0	‐	‐	‐	‐	‐	‐	‐	‐	‐	‐	‐	‐
CTV2	18.0	‐	‐	‐	‐	‐	‐	‐	‐	‐	‐	‐	‐
PTV1	48.0	72.0	≤63.0	≤7.2	‐	‐	‐	‐	‐	≥95	‐	‐	‐
PTV2	14.4	21.6	≤18.9	≤2.2	‐	‐	‐	‐	‐	‐	‐	‐	≥95
Rectum	‐	‐	‐	‐	‐	≤2	≤12	≤20	≤30	≤45	≤50	≤70	‐
Bladder	‐	‐	‐	‐	≤2	≤12	≤20	≤30	≤45	‐	≤60	‐	‐
Left Hip	‐	45.0	‐	‐	‐	‐	‐	‐	‐	‐	‐	‐	‐
Right Hip	‐	45.0	‐	‐	‐	‐	‐	‐	‐	‐	‐	‐	‐

IMPT plans were calculated on CT‐A1 using Varian Eclipse software version 8.6 (Varian Medical Systems, Palo Alto, CA). The isocenter was chosen to be the volumetric center of the prostate, as calculated from the prostate markers. A virtual machine with PBS abilities and an initial beam sigma of 0.4 cm in air was used. The spot spacing in and between scanning layers was 0.5 cm. The grid size used for the calculation was $0.25\times 0.25\times 0.25{\text{cm}}^{3}$. For each of the patients, four plan arrangements were considered. In this study, each plan consisted of two fields: Bi‐lateral (90° and 270°) and lateral—oblique (85° and 275°). The different beam angulations were considered to avoid beam paths parallel to interfaces of density changes as would be expected if the axis connecting the femur head and the greater and lesser trochanter structures is parallel to the proton beam. No distal modifications were performed to compensate for setup errors or range uncertainties. For each field arrangement, single field optimization (SFUD) and multiple field optimization (MF) were tested. The option of the field optimization technique was used as offered in the plan setup mode of the treatment planning system. To summarize, a bilateral MF (LMF), an oblique MF (OMF), a bilateral SFUD (LSF), and an oblique SFUD (OSF) treatment plans were calculated for each patient in this study. The dose to the different structures was analyzed using DVH parameters and generalized equivalent uniform dose (EUD)^(^ ^22^ ^)^ to help identify cold spots. EUD was evaluated as $\text{EUD}={\left[1/N{\sum_{i}^{N}\left(d_{i}\right)}^{x}\right]}^{1/\alpha }$, where *i* was the voxel of interest which receives a dose $d_{l}$, *N* was the total number of voxels, a was the tissue specific volume parameter. α of −10 was chosen for EUD calculation of the prostate.^(^ ^6^ ^,^ ^24^ ^–^ ^25^ ^)^

The effect of geometrical changes on dose distribution in the CTV and the OAR were analyzed separately for intra‐ and interfractional motion type. To analyze the effect of intrafractional motion on dose distribution, the treatment plans were recalculated on CT‐A2 and CT‐A3. Assuming that the patients will be perfectly positioned at the beginning of the first treatment fractions, CT‐A1, CT‐A2, and CT‐A3 can be considered as snapshots of patient anatomy at the beginning, half way, and at the end of a treatment fraction, respectively. Intrafractional interplay effects and anatomical changes between fields within a fraction were not analyzed. The interplay between position of spots of a PBS field and anatomical changes is beyond the scope of this work.

In analyzing interfractional motion, the dose is recalculated on the CT‐B1 which was registered to CT‐A1 using bony landmarks. Then, the field's isocenter was readjusted to match the volumetric center of the prostate as calculated from the prostate markers. The correction in the position of the isocenter was equivalent to translational setup corrections of patient position between fractions.

CTV coverage was considered the criterion for plan robustness. A plan was deemed robust if the minimum dose in CTVs was more than 90% of the prescribed dose (54.0 ${\text{Gy}}_{1.1}$ for CTV1 and 70.2 ${\text{Gy}}_{1.1}$ for CTV2) in CT‐A2, CT‐A3, and CT‐B1. Meanwhile, the effect of change in patient anatomy on the dose to the OAR was analyzed using DVH parameters.

## III. RESULTS

Table 2 shows the different setups of patient immobilization and the consequent inter‐ and intrafractional motion. Looking at the changes in bony anatomy, two distinct patterns of motion can be distinguished. One is the translation and rotation of the pelvic girdle. The other is the rotation of the femur. The position of the femoral heads is fixed with respect to the pelvic girdle even when a rotation of the femur is observed. However, such a rotation results in a change in the alignment of the femoral neck and the adjacent greater and lesser trochanter structures. The quantification of changes in bony anatomy is not trivial. From the data, one out of six patients (17%) who were immobilized with the half body forms showed intrafractional motions of the bony anatomy. In comparison, three out of four patients (75%) in the knee–foot fixation showed change in the bony anatomy intrafractionally. Interfractionally, change in bony anatomy happens in higher frequency in patients with knee–foot fixation (50%) vs. patients in the form (33%).

**Table 2:** Summary of intra‐ and interfractional motion of the study patients. The reported values are all relative to the initial planning CT (CT‐A1). If no value is given, the observed change was less than 3 mm.

*Patient Index*	*Immobilization Setup*	*Change in Bony Anatomy*	*Presence of Rectal Balloon*	*Change in Rectal Balloon Position in CT‐B1* ^a^	*Change in Bladder Volume in CT‐A2*	*Change in Bladder Volume in CT‐A3*	*Change in Bladder Volume in CT‐B1* ^b^
1	Form		No		18%	45%	9%
2	Form	Interfractional	No		0%	0%	−15%
3	Form	Interfractional	No		4%	7%	−67%
4	Form		No		8%	18%	−15%
5	Knee‐foot		No		6%	10%	−5%
6	Knee‐foot	Intra‐ and Interfractional	No		12%	27%	−38%
7	Knee‐foot	Intra‐ and Interfractional	No		10%	34%	−75%
8	Form		Yes	−1.0cm	0%	11%	93%
9	Knee‐foot	Intrafractional	Yes		12%	27%	−21%
10	Form	Intrafractional	Yes	−3.5cm	8%	20%	23%
Average±SD					8±6%	20±14%	−11±48%

a The changes in position of rectal balloon are reported only along the crania–caudal direction.

b Summary of the average ± standard deviation of the change in bladder volume.

In two out of three patients who received a rectal balloon, changes exceeding 0.5 cm in the position of the balloon were observed in the repeated CTs (see Table 2). The Table also summarizes measured change in bladder volume in different CTs relative to CT‐A1 where a positive change indicates increase in bladder volume.

In Table 3, the change in position of the prostate relative to CT‐A1, calculated from the internal markers, is shown for all three directions: Left–Right (LR), Anterior–Posterior (AP) and Cranio–Caudal (CC). Furthermore the amplitude of the 3D vector is also calculated.

**Table 3:** Change in position of the prostate as calculated from the internal markers relative to CT‐A1; all values are presented in cm. The amplitude of the 3D vector is also shown, along with the average and standard deviations (SD).

			*CT‐A2*				*CT‐A3*				*CT‐B1*	
*Patient Index*	*LR*	*AP*	*CC*	*3D Vector*	*LR*	*AP*	*CC*	*3D Vector*	*LR*	*AP*	*CC*	*3D Vector*
1	0.0	0.1	0.0	0.1	−0.1	0.1	0.0	0.1	−0.1	−0.1	−0.2	0.2
2	0.1	0.2	0.0	0.2	0.0	−0.1	−0.1	0.1	0.0	0.3	−0.1	0.3
3	−0.1	−0.1	0.2	0.2	0.0	−0.2	0.2	0.2	0.0	−0.1	0.1	0.1
4	0.1	0.0	0.2	0.2	0.1	−0.1	0.3	0.3	0.0	−0.3	0.3	0.4
5	0.0	−0.4	0.5	0.6	0.0	−0.2	0.2	0.3	−0.2	−0.6	0.7	0.9
6	−0.1	0.2	−0.4	0.4	0.1	0.2	−0.3	0.4	−0.6	−0.3	0.3	0.8
7	0.0	−0.1	−0.1	0.1	0.0	0.2	−0.3	0.3	0.1	0.0	0.2	0.2
8	−0.1	−0.1	−0.2	0.2	0.2	0.2	−0.3	0.4	0.1	−0.1	−0.2	0.2
9	0.1	0.0	0.2	0.2	0.0	−0.1	0.0	0.1	−0.1	−0.6	0.5	0.8
10	0.1	−0.2	0.3	0.4	0.0	−0.7	0.3	0.8	0.2	−0.6	0.2	0.7
Average	0.0	0.0	0.1	0.3	0.0	−0.1	0.0	0.3	−0.1	−0.2	0.2	0.5
SD	0.1	0.2	0.3	0.2	0.1	0.3	0.2	0.2	0.2	0.3	0.3	0.3

LR=Left‐Right; AP=Anterior‐Posterior; CC=Cranio‐Caudal.

**Figure 1:**
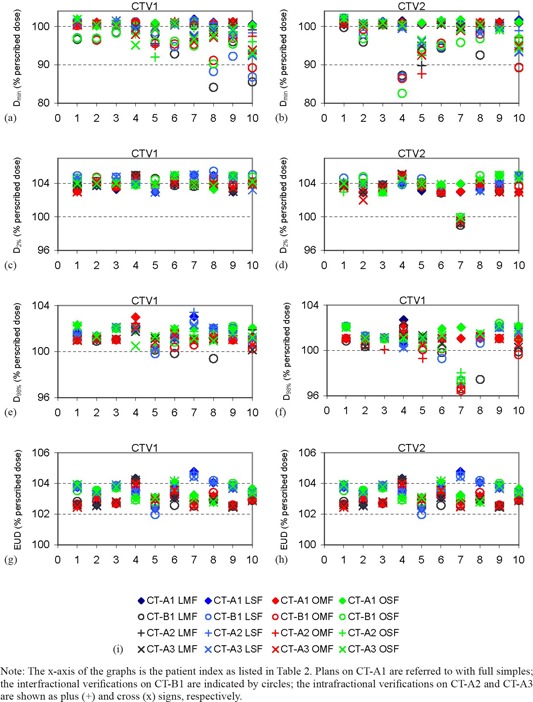
$D_{\text{min}}$, $D_{98\%}$, $D_{98\%}$, and EUD for CTV1 and CTV2 ((a)–(h)); legend (i) for all graphs. Note: The x‐axis of the graphs is the patient index as listed in Table 2. Plans on CT‐A1 are referred to with full simples; the interfractional verifications on CT‐B1 are indicated by circles; the intrafractional verifications on CT‐A2 and CT‐A3 are shown as plus (+) and cross (x) signs, respectively.

All initial treatment plans (optimized based on CT‐A1) fulfilled the treatment planning criteria in Table 1 in terms of CTV and PTV coverage, as well as OAR sparing. An exception was the fourth patient for whom the PTV2 enclosed 11% of the rectal volume. There, it was geometrically impossible to fulfill the $V_{75}$ constraint in that case. Comparing the bilateral plans with the oblique field arrangement (OMF vs. LMF and OSF vs. LSF) and comparing multiple field optimization vs. single field optimization (LMF vs. LSF and OMF vs. OSF), no significant changes in the EUD of CTVs was observed. The EUD was always 2.5%−3.5% higher than the prescribed dose. Similarly, $D_{\text{min}}$, $D_{98\%}$ and $D_{2\%}$ showed little variation due to selection of planning technique for the initial treatment plans. Effects due to changes in patient anatomy were most prominent in $D_{\text{min}}$ compared to the other parameters (see Fig.1).

As for robustness, $D_{\text{min}}$ in CTVs was more than 90% of the prescribed dose in CT‐A2, CT‐A3 (intrafractionally), and CT‐B1 (interfractionally) in most patients (see Fig.1). All plans of all patients are robust with respect to intrafractional motion, except for the fifth patient. In that case, the position of the prostate varied by a 3D vector of 0.6 cm. The LSF and OSF plans were above the 90% constraint and, therefore, robust. Interfractionally, patient 4 fails the criterion on $D_{\text{min}}$ with all modalities. However, no major motion patterns were detected interfractionally in the case of patient 4. Upon close inspection, the CTV2 volume which received 100% of the prescribed dose ($V_{100\%}$) was found to vary between 99.8% and 99.9% suggesting that only 0.1%−0.2% of the voxels were represented by $D_{\text{min}}$ values. Patients 8 and 10 are not robust to interfractional motion. In both cases, the rectal balloon was used, but the balloon was not placed at the same position as in CT‐A1. Nonetheless, both LSF and OSF techniques for patient 8 and the OSF of patient 10 were still robust. Meanwhile, the near minimum value ($D_{98\%}$) is above the 96% and the near maximum ($D_{2\%}$) is less than 106% for all patients (see Fig.1). $D_{98\%}$ appears insensitive to the changes in anatomy compared to $D_{\text{min}}$.

**Figure 2:**
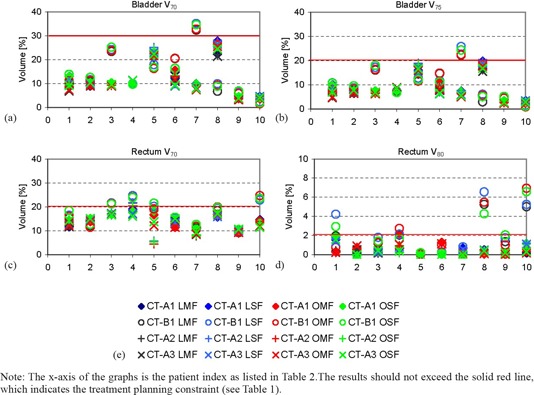
Bladder $V_{70}$ and $V_{75}$, as well as Rectal $V_{75}$ and $V_{80}$, ((a)–(d)) for different treatment planning techniques using the initial and verification CT images; legend (e) for all graphs. Note: The x‐axis of the graphs is the patient index as listed in Table 2. The results should not exceed the solid red line, which indicates the treatment planning constraint (see Table 1).

For bladder and rectum, Fig.2 displays some of the controlled volume parameters on CT‐A1 and the verifications on CT‐A2, CT‐A3, and CT‐B1. Logically, an increase in the volume causes a reduction of the measured dose parameters. For most patients, bladder dose‐volume parameters are far beyond the tolerance values set in Table 1 for the initial treatment plans. The values are within tolerance for all intrafractional verifications. In the case of the 7th patient, a reduction of the bladder volume by 75% led to exceeding the tolerance on $V_{70}$ and $V_{75}$ in all interfractional verifications.

For rectal dose parameters, the fourth patient was the only patient for which rectal doses exceeded the tolerances intrafractionally. Interfractionally, in the cases where the repositioning of a smaller rectal balloon of 50 ml was performed, rectal balloons showed a large variation — $V_{80}$ was 2–4 fold the accepted tolerance volume of 2%. These changes were also reflected on $D_{\text{min}}$ of CTV1 for both patients 8 and 10. Smaller effects were seen in CTV2 (see Fig.1).

## IV. DISCUSSION

Table 2 shows the different patterns of patient positioning and the consequent inter‐ and intrafractional change in bony anatomy. The change in bony anatomy results in changes in the path length of the protons and will degrade the optimized dose distribution. Therefore these changes should be minimized in patients for proton and ion therapy. With the help of image‐guidance techniques, changes in position or alignment of the pelvic girdle are easily detected and corrected. On the other hand, corrections for rotations in the upper thigh bones with respect to the pelvic girdle are less successful. Such corrections are only performed interfractionally. Nonetheless, both types of motion can be observed intra‐ and interfractionally. The rotation of the upper thigh bones with respect to the pelvic girdle is usually hindered by the immobilization setup. Meanwhile, if the patient relaxes in the immobilization setup or moves within, changes in the bony anatomy can still be observed. Effective patient immobilization restricts intra‐ and interfractional motion. The results of our study suggest that a body form provides better fixation than the knee–foot fixation, especially intrafractionally.

Rectal balloons are used to reduce rectal toxicity during treatment and improve patient positioning intrafractionally. However, in two out of three cases, the placement of the rectal balloon was different from the position recorded in the initial planning CT. But as the catheters are not marked or indexed, interfractional displacement in the CC direction is likely to happen.^(^ ^17^ ^)^ The effect of setup errors in the rectal balloon is insufficient dose coverage in CTV1 for both patients 8 and 10. The effect on CTV2 was less significant than on CTV1. This is due to the fact that CTV1 includes both the prostate and the seminal vesicles, while CTV2 includes mainly the prostate and a less fraction of the seminal vesicles than CTV1. The seminal vesicles are less rigid than the prostate and surround the anterior rectal wall. Hence, the structure of the seminal vesicles deforms in shape with the change in rectal filling, while the prostate mostly suffers translational and rotational shifts. The effect of translation shifts in the prostate itself can be verified and corrected for using the markers. However, the deformation in the seminal vesicles could not be corrected. In this work, the CTV to PTV margin was 0.8 cm around the seminal vesicles. Further expansion of the PTV margin around the seminal vesicles can perhaps ensure interfractional dose coverage of CTV1. The authors also suggest using long rectal balloons with volume capacity over 100 ml in order to stabilize the seminal vesicles. It was also found that multiple field optimizations were less robust than the single field optimization in both patients. In the robustness test of $D_{\text{min}}$ for CTV1, five plans (3 MF and 2 SF) failed the test with $D_{\text{min}}$ < 54.0 ${\text{Gy}}_{1.1}$. For CTV2, eight plans (6 MF and 2 SF) failed with $D_{\min}<70.2$ ${\text{Gy}}_{1.1}$. The weights of the spots per field are calculated independently for each field in SF optimization. Hence, the target voxel is irradiated by each field. Thus, if one field is affected by organ motion, the other can possibly recover the dose distribution.

Due to the systematic correction of the isocenter, dose conformity in the CTVs is restored despite large shifts in the prostate positions. However, if the shifts occur intrafractionally, they are not corrected. In most cases, the 3D amplitude of the shift is less than the 0.5 cm CTV to PTV margin despite the long time involved (~5min). This agrees with the results of Vargas et al.^(^ ^11^ ^)^ In their work, shifts by up to 0.5 cm have small effects of the CTV coverage using a similar CTV to PTV margin. In cases where the shift was larger than 0.5 cm (0.6 cm in patient 5), multiple field optimization plans were not robust. In the case of patient 5, no major changes in bony anatomy were detected (see Table 2). The recorded shift indicates that the target volume simply moved away from the isocenter by 0.6 cm.

In all the plans and verifications, the EUD of CTV1 and CTV2 were always higher than the prescribed dose due to the tight constraints on CTV and PTV values during treatment planning optimization.

Using oblique fields did not have a significant effect on the dose distribution of most patients when compared to bilateral fields. Comparison of $D_{\text{min}}$ (see Fig.1) shows that SFUD methods were generally more robust than multiple field optimizations. However, the location of the $D_{\text{min}}$ values is dependent on the deformation of the patient, and is likely to average out during the course of a multifraction treatment series.

## V. CONCLUSIONS

The presented cases demonstrated a range of intra‐ and interfractional motion expected in prostate cases, and their dosimetric consequences. Interfractional motion can be reduced using accurate patient positioning and image guidance. When large motions were observed, SFUD plans appear more robust than MF plans. Meanwhile, one or more robust proton plans using PBS was found despite the different intra‐ and interfractional motion patterns and different immobilization setup of the patients.

In ongoing studies, several models of rectal balloons are being tested with respect to repositioning accuracy and robustness of proton plans, and a wider spectrum of field arrangements are being investigated.
